# Manipulation of current rectification in van der Waals ferroionic CuInP_2_S_6_

**DOI:** 10.1038/s41467-022-28235-6

**Published:** 2022-01-31

**Authors:** Xingan Jiang, Xueyun Wang, Xiaolei Wang, Xiangping Zhang, Ruirui Niu, Jianming Deng, Sheng Xu, Yingzhuo Lun, Yanyu Liu, Tianlong Xia, Jianming Lu, Jiawang Hong

**Affiliations:** 1 School of Aerospace Engineering, Beijing Institute of Technology, 100081 Beijing, China; 2 College of Physics and Optoelectronics, Faculty of Science, Beijing University of Technology, 100124 Beijing, China; 3 State Key Laboratory for Mesoscopic Physics and Frontiers Science Center for Nano-optoelectronics, School of Physics, Peking University, 100871 Beijing, China; 4 Department of Physics and Beijing Key Laboratory of Opto-electronic Functional Materials & Micro-nano Devices, Renmin University of China, 100871 Beijing, China; 5 College of Physics and Materials Science, Tianjin Normal University, 300387 Tianjin, PR China

**Keywords:** Electronic properties and materials, Ferroelectrics and multiferroics

## Abstract

Developing a single-phase self-rectifying memristor with the continuously tunable feature is structurally desirable and functionally adaptive to dynamic environmental stimuli variations, which is the pursuit of further smart memristors and neuromorphic computing. Herein, we report a van der Waals ferroelectric CuInP_2_S_6_ as a single memristor with superior continuous modulation of current and self-rectifying to different bias stimuli (sweeping speed, direction, amplitude, etc.) and external mechanical load. The synergetic contribution of controllable Cu^+^ ions migration and interfacial Schottky barrier is proposed to dynamically control the current flow and device performance. These outstanding sensitive features make this material possible for being superior candidate for future smart memristors with bidirectional operation mode and strong recognition to input faults and variations.

## Abstract (web summary)

Developing a single-phase self-rectifying memristor is desirable and functionally adaptive to dynamic environmental stimuli variations. Here, the authors report a single phase CuInP_2_S_6_ based memristor with continuously tunable current rectifying.

## Introduction

Recent advances in controlling the ionic migration processes in solid-state thin films have led to the rapid development of ionic functionalities^1,2^. One representative application is ionic memristors for neuromorphic computing^3–5^, whereas the main obstacle is the undesired current path flowing through neighboring memory cells in the crossbar array architecture, resulting in write/read inaccuracy and unnecessary energy consumption^6^. To overcome this issue, ionic memristors must possess a high current rectification or nonlinearity, which can be solved by artificially connecting a series of transistors^7^, diodes^8^, or nonlinear selectors^9^ for constructing self-nonlinearity/self-rectifying. However, these complex methods inevitably bring sophisticated micro-nano fabrication/integration, low stacking density, and incompatible 3D vertical crossbar array under certain circumstances. Therefore, developing a single memristor system with self-rectifying characteristics is desirable due to its simplicity, but the progress towards this goal is hampered to a large extent by a lack of a suitable material.

Hitherto, the research of single memristors mainly focuses on oxide-based multicomponent layers by tuning oxygen defects or a single insulating material by grafting the Ag filament. The former excessively depends on a multilayer interface and inevitably suffers from the current leakage from oxygen defects^10,11^. The latter needs assistance by doping fast diffusive silver ions or using active silver electrodes^12,13^. It’s also worth mentioning that the current developed ionic memristors usually show a unidirectional rectifying direction. For example, in Ag-based memristors, the relatively abrupt set process makes it difficult to continuously control the conducting states and current rectifying direction as a response to external stimuli^14^. However, these dynamic tunable features are critical for bidirectional operation mode and environment-adaptable learning through the neural network as a response to dynamic environmental stimuli. Therefore, a single-phase memristor simultaneously with highly mobile ions and continuously tunable feature is in urgent need of exploration.

Electric field tuning of physical properties in Van der Waals materials is a very lively field^15–17^. In recent years, the memristor behavior of 2D van der Waals material has also aroused great attentions^18–20^. One veritable star compound from the thio/selono-phosphate family, CuInP_2_S_6_ (CIPS), meets the requirements for a tunable single-phase memristor from the following three advantages. (1) An identifying feature of this material is the electrochemically active Cu^+^ ions in lattice, which endows itself with an excellent insulating property at a low voltage, and exhibits highly sensitive response of current to electric stimuli above a threshold voltage^18,21^. (2) Due to the van der Waals interaction, CIPS can be easily exfoliated and transferred to desired substrates. (3) Current developed ionic memristors are mostly based on metallic oxide materials. These materials are usually rigid and non-piezoelectric, which are insensitive to external mechanical stimuli. On the contrary, CIPS material (Cc phase) is the only discovered 2D van der Waals ferroionic material so far^22^, and possesses good flexibility and piezoelectric characteristic^22,23^, which makes it sensitive to the external strain/strain gradient field.

Taking above mentioned advantages, in this work, we study the ionic migration of Cu^+^ and current rectifying behavior in single-phase CIPS materials by using conductive scanning tips to apply different bias and mechanical stimuli. A 120-nm-thick CIPS single material shows a high ON/OFF ratio of ~10^3^ along with a self-rectifying ratio of ~10^3^. More importantly, this material possesses an excellently continuous modulation of current and rectifying direction by different bias (including the amplitude, speed, and direction) and mechanical strain, which is physically based on the synergetic contribution of controllable Cu^+^ ions migration and interfacial Schottky barrier. These outstanding sensitive features make this material possible for being superior candidate for smart memristors with bidirectional operation mode and strong recognition to input faults and variations.

## Results

**Fig. 1:**
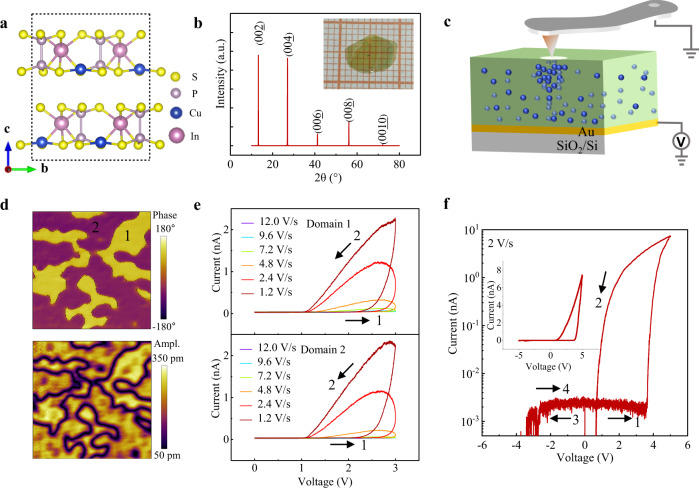
Characterizations of CIPS single crystal and its electrical properties. **a** CuInP_2_S_6_ crystal structure with a vdW gap between the layers; **b** The measured XRD pattern for as-grown CIPS single crystal with ab crystalline plane, the inset shows the flexible CIPS crystal. **c** The schematic experimental setup for electrical measurement. Cu^+^ ions are represented by the blue balls, which undergoes directional migration driven by the external electric field. **d** Out of plane PFM phase and amplitude images of CIPS flakes, respectively; **e** The consecutive sweeping speed-dependent *I*–*V* semi-cycles (0 V→+ 3 V→0 V) measured on two different domains with opposite direction in **d. f** A full cycle of *I*–*V* curve by sinusoidal voltage sweeping of 0 V→5 V→-5 V→0 V at a sweeping speed of 2 V/s. The thickness of CIPS is 120 nm.

The crystal structure of CuInP_2_S_6_ can be described as a sulfur framework in which metal cations (Cu and In) and P−P pairs fulfill the octahedral voids, as shown in Fig. 1a. In this system, the charged Cu^+^ ions become mobile across the lattice periodic potentials under a high electric field, which is the fundamental of ionic conductivity^24^. The as-grown CIPS single crystal shows a natural *ab*-plane. The sample quality is characterized by performing XRD and SEM-EDS experiments, as shown in Fig. 1b and Supplementary Fig. 1, respectively. For the electrical measurements, CIPS nanoflakes were mechanically exfoliated and subsequently taped on conductive Au electrode (see the details in Methods Section). The schematic experimental setup is shown in Fig. 1c, the conductive scanning tip is utilized as the top nanoelectrode, which facilitates the manipulation of a localized conductive path^14,25^. The voltage was applied to the bottom Au electrode and all the electrical measurements in our experiments were conducted on 120-nm-thick CIPS nanoflakes, see Supplementary Fig. 2.

Given the slow mobility of ions, ions migration can be more reliably controlled by the sweeping speed of electric field. Continuous modulation of current by changing the voltage sweeping speed was demonstrated. A series of semi-cycle of *I*–*V* curves on two different ferroelectric domains (Fig. 1d) were consecutively measured by gradually reducing the sweeping speed from 12.0 V/s to 1.2 V/s, and the corresponding results are shown in Fig. 1e. When the voltage is swept forward from 0 V to 3 V with fast sweeping speed (>4.8 V/s), CIPS is in a high insulating state, so called current-off state. As the sweeping speed decreases below 4.8 V/s, the current off-on transition occurs at a certain threshold voltage and the current increases sharply with a lower sweeping speed, which produces a clear current hysteresis as the voltage is swept backward from 3 V to 0 V. The higher current at a slower sweeping speed (conduction activation) is a fingerprint of ionic conductors/memristors^18^, as a slow sweeping speed allows Cu^+^ ions to migrate sufficiently. The cross-sectional SEM-EDS measurement further supports the electrical field-driven Cu^+^ immigration, as shown in Supplementary Fig. 3. Note that the *I*–*V* results on two different domains show no obvious difference, ruling out the effect of the ferroelectric polarization and further highlighting the dominant role of the mobile Cu^+^ ions in such sweeping speed-controlled current and current hysteresis. When the voltage is swept from 0 V to 5 V at the speed of 2.0 V/s, the current-off state is switched to current-on state at a threshold voltage of ~3.8 V due to the activated Cu^+^ ions migration, which contributes to a high ON-OFF ratio of ~10^3^ at 2.5 V as the voltage is swept backward from 5 V to 0 V. When the voltage is swept from 0 V to -5 V, almost no current flows is detected due to the rectifying function of the interfacial Schottky barrier, which contributes to a self-rectifying ratio (I_+2.5V_/I_−2.5V_) of ~10^3^ at 2.5 V (Fig. 1f). Note that a negative voltage reconfigures the distribution of Cu^+^ ions and resets the high conducting state into the insulating state again, leading to the repeatable full-cycle of *I*–*V* curves as shown in Supplementary Fig. 4.

**Fig. 2:**
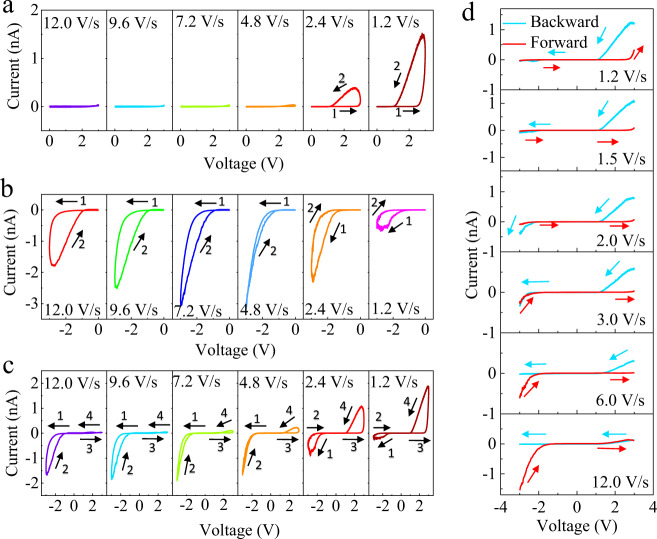
Current rectification modulated by the sweeping speed/direction. **a**–**c** Response of current–voltage (*I*–*V*) curves to sweeping speed with a sinusoidal voltage sweeping semi-cycle of (0 V→+ 3 V→0 V), (0 V→−3 V→0 V), and full-cycle of (0 V→−3 V→+ 3 V→0 V), respectively; *I*–*V* curves are consecutively measured at a gradually lower sweeping speed ranging from 12.0 V/s to 1.2 V/s. The numbers 1-4 denote the sweeping sequence. **d** Response of *I*–*V* curves measured by the forward voltage sweeping (−3 V→+ 3 V) and backward voltage sweeping (+3 V→−3 V) at a different sweeping speeds ranging from 12.0 V/s to 1.2 V/s on two adjacent sites.

The sweeping speed-modulated current rectification was further investigated with different sweeping frequencies and opposite voltage directions. As shown in Fig. 2a, in the semi-cycle with a positive voltage, a slow sweeping speed, i.e. 2.4 V/s, is required to initiate the Cu^+^ ions migration accompanied by the current boost and hysteresis, which can be further enhanced by a slower sweeping speed of 1.2 V/s. However, the current shows a strikingly different response in the semi-cycle with negative voltage, as shown in Fig. 2b. It is clearly observed that the current boosts at a sweeping speed of 12.0 V/s, continuously increases until reaching a “peak value” at the speed of 4.8 V/s, and then starts a dramatic decline. The current hysteresis also shows changes in response to a slower sweeping speed, with its gap continuously reducing and almost disappearing at the sweeping speed of 4.8 V/s, and then being reversed in the direction. The different current response to the sweeping speed in opposite voltage direction makes it possible for the sweeping speed-controlled current rectification. As shown in Fig. 2c, a clear sweeping speed-modulated current rectification is observed, more specifically, the preferential current flow in negative voltage is inversed to positive bias direction as the voltage is swept from 12.0 V/s to 1.2 V/s. The full-cycle of *I*–*V* curves with opposite voltage sweeping are also measured and reproduce a similar result (Supplementary Fig. 5). In addition, we conducted a similar electrical measurement using the top macroscopic platinum electrode (1 × 1 μm^2^), as shown in Supplementary Fig. 6, which reproduces the control of current rectifying behavior and further rules out the possible dominating effect of electrochemical reaction with atmosphere in the c-AFM measurement. The *I*–*V* curves with the consecutively forward (−3 V→+ 3 V) and backward sweeping (+3 V→−3 V) also reveal the function of self-rectifying (Fig. 2d), i.e, with 6.0 V/s sweeping speed, the forward and backward sweeping induces the current flow in the reversed direction. In addition to the sweeping speed-controlled self-rectifying, the magnitude of sweeping voltage and sweeping cycles can also tune the self-rectifying, as shown in Supplementary Figs. 7 and 8. Due to the nature of the time-dependent Cu^+^ immigration, pulse frequency also modulates the current rectifying behavior similar to the sweeping speed, as shown in Supplementary Figs. 9 and 10. As the pulse frequency increases, the current in the positive voltage starts to increase, however, the current in the negative voltage starts to decrease, which reverses the direction of current flow. The sensitivity of current and rectifying behavior to sweeping parameters and pulse frequency manifests its potential application in future neurofunction in response to complex environmental conditions.

**Fig. 3:**
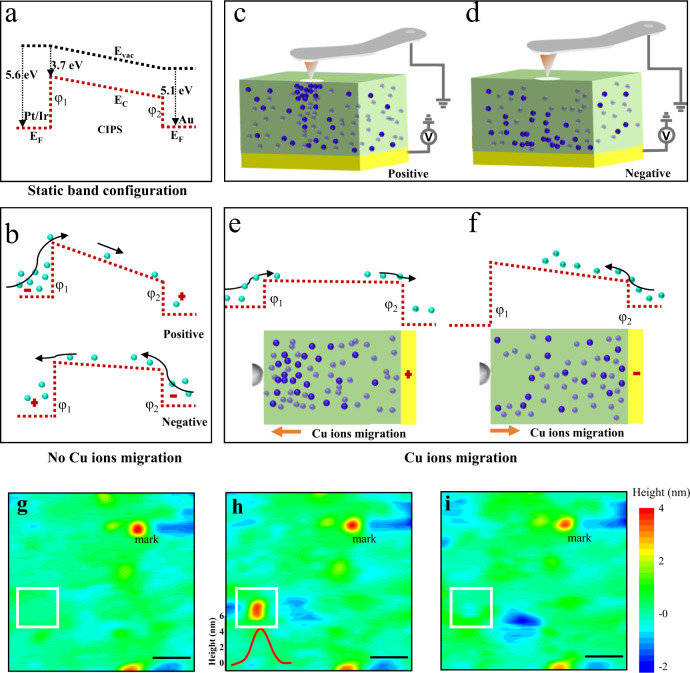
Physical mechanism of highly mobile Cu^+^ ions-meditated current rectification. **a** Schematic static band configuration for tip/CIPS/Au bottom electrode measurement setup according to the work function of Pt/Ir tip (5.6 eV)^42^, Au (5.1 eV)^43^, and electron affinity of CIPS (3.7 eV)^44^. The difference in the work function/electron affinity creates asymmetric interfaces, with the Schottky barrier *φ*_1_ and *φ*_2_ at the side of Pt/Ir tip and the bottom Au electrode, respectively; **b** In the absence of Cu^+^ ions migration, the schematic electrons (cyan balls) transport process in different bias direction, where the bias voltage is applied to the bottom Au substrate; **c**, **d** Cu^+^ ions (blue ball) undergo directional migration driven by the applied voltage. **e**, **f** In the presence of Cu^+^ ions migration and redistribution, the schematic Cu^+^ ions (blue balls) migration and electrons (cyan balls) transfer for current flow. **g**–**i** The topography image in virgin state, after two consecutive positive voltage sweeping semi-cycle (0 V→+4 V→0 V), and after two consecutive negative voltage sweeping semi-cycle (0 V→−4 V→0 V), respectively. The *I*–*V* measurements are conducted in the white square area in **g**–**i** and the ever-present bump in the mark location rules out the possibility of tip scraping for the bump elimination during scanning. The scale bar length is 200 nm in **g**–**i**.

In general, current rectifying behavior usually occurs at asymmetric interfaces such as *p-n* junctions or Schottky barriers, where electrons and holes are the carriers of the entire system^26^. However, in ionically active CIPS materials, both Cu^+^ ions and electron carriers participate in the electrical transport. The slow redistribution process of Cu^+^ ions under the electric field can be reliably controlled by the sweeping speed, which is the key to revealing the underlying mechanism for the sweeping speed-controlled self-rectifying behavior. From the energy band configuration, as sketched in Fig. 3a, the difference in the work function/electron affinity creates asymmetric interfaces, with the Schottky barrier *φ*_1_ and *φ*_2_ at the side of Pt/Ir tip and the bottom Au electrode, respectively. In the absence of Cu^+^ ions migration, as shown in Fig. 3b, the electrons need to overcome a larger barrier *φ*_1_ under positive voltage and a smaller barrier *φ*_2_ under negative voltage for current flow, which leads to smaller voltage drops across the CIPS under positive voltage. In the presence of Cu^+^ migration, the Cu^+^ ions will undergo directional migration driven by the electric field and generate a localized conducting path under the tip, as shown in Fig. 3c, d. The fast/slow sweeping speed corresponds to the case of weak/strong Cu^+^ migration, respectively. In positive voltage sweeping as shown in Fig. 2a, almost no current flow is detected at a sweeping speed faster than 4.8 V/s due to the larger interface barrier *φ*_1_ and weak Cu^+^ migration. As sweeping speed decreases below 4.8 V/s, the current increases sharply due to the enhanced Cu^+^ migration. Note that more Cu^+^ ions migrate toward and concentrate around the tip in this process and generate a “Cu^+^ ions-rich” region, which reduces the Schottky barrier *φ*_1_ under the tip via an ionic capacitive effect^27,28^ or electrochemistry reaction^29^, as shown in Fig. 3e. The reduction of Schottky barrier *φ*_1_ further synergistically boosts the current by distributing more voltage drops on CIPS to enhance the Cu^+^ ions migration. In negative voltage sweeping as shown in Fig. 2b, although at a fast sweeping speed of 12.0 V/s, Cu^+^ ions migration can be easily initiated due to the lower interface barrier *φ*_2_, which boosts the current. However, as shown in Fig. 3f, Cu^+^ ions are also expelled from the tip in negative voltage sweeping to generate a “Cu^+^ ions-deficient” region under the tip and imposes a higher potential barrier *φ*_1_. In this case, the competition between Cu^+^ ions migration and the interfacial Schottky barrier change exists in negative voltage sweeping, which depends on the sweeping speed. As the sweeping speed decreases below 4.8 V/s, it is difficult to continuously drive Cu^+^ ion migration in the bulk, leading to the dramatic decline in current accompanied by a reversal of hysteresis, as shown in Fig. 2b. The migration of Cu^+^ ions can be also verified from the topographic changes^30^, as shown in Fig. 3g–i, where a slightly topographic bump (~5 nm) appears with *I*–*V* measurement in the white square area after positive voltage sweeping, and can be reversibly eliminated by the subsequent negative voltage sweeping.

**Fig. 4:**
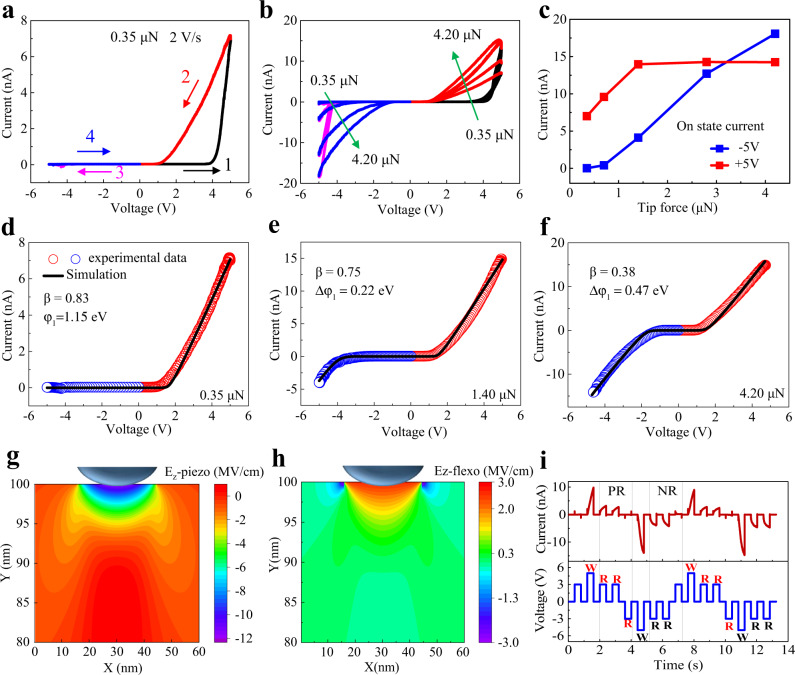
The mechanical modulation of current rectifying in CIPS via the AFM tip force. **a** The *I*–*V* curves with a voltage sweeping of 0 V→+ 5 V→−5 V→0 V measured at a sweeping speed of 2 V/s. The number 1-4 denotes the sweeping sequence in a full cycle. The initial tip compressive force of 0.35 μN is set to the contact force between tip and nanoflakes. **b** The *I*–*V* curves with a voltage sweeping (0 V→+5 V→−5 V→0 V) at a sweeping speed of 2 V/s, but with different tip compressive force: 0.35 μN, 0.70 μN, 1.40 μN, 2.80 μN, 4.20 μN. **c** The current in ON state at ±5 V versus tip force. **d**–**f** The *I*–*V* data in ON state in positive (red) and negative voltage (blue) at different tip force is simulated (black line) by the modified thermionic emission theory; **g**, **h** the piezoelectric field (E_z_-piezo) and flexoelectric field (E_z_-flexo) in CIPS flakes obtained from theoretical calculations with a tip-force model under a tip force of 4.20 μN, respectively. **i** A consecutive write (±5 V) and read (±3 V) voltage pulses measurement at a constant tip force of 4.20 μN. A large positive/negative voltage pulse in writing operation leads to the positive/negative rectification (PR/NR) in the small voltage reading operation.

In addition to the sweeping speed-controlled current rectifying, the mechanical modulation of current rectifying is also revealed. As a simple demonstration, the local strain/strain gradient is applied by the scanning tip. With a small tip force of 0.35 μN as the contact force, the *I*–*V* curve shows unidirectional current flow in positive voltage direction, as shown in Fig. 4a. As the tip force is gradually increased to 4.20 μN (Fig. 4b), the current under positive voltage gradually increases and eventually reaches its saturation value above 1.40 μN, while the current under negative voltage increases rapidly and even exhibits a larger value than that under positive bias above 2.80 μN. The current dependence on tip force in ON state at ±5 V is shown in Fig. 4c. The bidirectional current-on state can be repeatedly reset to current-off state by a reverse voltage, which resembles a switchable current rectifying. The modified thermionic emission theory is further used to describe the interfacial electron injection in the case of Cu^+^ ions migration (see the details in Supplementary Figs. 11 and 12). Here we introduce a *β* factor to describe the influence of the Cu^+^ ions migration and the modified I-V rectifying behavior. The value of *β* = 0, <0.5 and >0.5 represents the case of no Cu^+^ ions migration, a stronger Cu^+^ ions migration in negative voltage direction, and a stronger Cu^+^ ions migration under positive bias, respectively. Δ*φ*_1_ represents the Schottky barrier change under the mechanical load. The I-V data in bidirectional current-on state in Fig. 4b was simulated with modified thermionic emission theory. As shown in Fig. 4d–f, as the tip force increases from 0.35 μN to 4.20 μN, *φ*_1_ decreases with a value of Δ*φ*_1_ = 0.47 eV, which synergistically enhances the Cu^+^ ions migration under negative voltage by distributing more voltages drops on CIPS. This phenomenon is manifested by the *β* factor decreasing from 0.83 to 0.38. Note that tip force-induced bandgap or contact area change is not the dominating factor, as shown in Supplementary Figs. 13 and 14.

Herein, the Schottky barrier change is probably related to strain and strain gradient under the AFM tip^23,31^, which induces an additional electric field from the piezoelectric effect and flexoelectric effect (Fig. 4g, i) and therefore synergetically reduces the Schottky barrier under the tip. The calculated strain and strain gradient are shown in Supplementary Fig. 15. The induced surface potentials from these two effects are shown in Supplementary Fig. 16. The effective surface potentials for piezoelectric and flexoelectric effect near the tip are 1.06 V and -0.56 V, respectively, and together give a net surface potential of ~0.5 V, which is close to the Schottky barrier change of ~0.47 V under the tip force of 4.20 μN. Finally, we conducted the consecutive writing (±5 V) and reading (±3 V) bias pulse measurement at a tip force of 4.20 μN, as shown in Fig. 4i. The bidirectional threshold memory with self-rectifying function is manifested in the ability to control the current flow, where a large positive/negative voltage pulse in writing operation leads to the positive/negative rectification (PR/NR) in the small voltage reading operation. This dynamic characteristic of bidirectional threshold memory is critical for bidirectional operation mode and environment-adaptable learning through the neural network as a response to dynamic environmental stimuli.

## Discussion

In summary, we report a single-phase memristor based on van der Waals material, CuInP_2_S_6_ (CIPS), which satisfies continuously tunable electric features. An identifying feature of this material is the electrochemically active Cu^+^ ions, which endows CIPS with a superior continuous modulation of current and self-rectifying by the sweeping speed of voltage, voltage direction, voltage amplitude, etc. or mechanical strain. The synergetic contribution of controllable Cu^+^ ions migration and interfacial Schottky barrier change is proposed to control the current flow in the bulk as well as the injection-limited electrode contact. The dynamically tunable feature is in urgent demand for further evolution of smart memristors with bidirectional operation mode and strong recognition to input faults and variations.

## Methods

### Sample preparation

CIPS Single crystals were synthesized by the chemical vapor transport method using stoichiometric elemental precursors^32,33^. The precursors were sealed inside a vacuumed quartz ampoule and then subjected to a two-zone horizontal tube furnace for a reaction time of 212 hours, the hot zone and cold zone were set to 650 and 600 °C, respectively. The thin flakes were obtained by mechanical exfoliation, where the as-grown single crystal was first attached to the adhesive tape, then repeatedly folded and tore off, and finally transferred to the conductive Au (5 nm)/SiO_2_(285 nm)/Si substrate. Au was deposited by electron-beam evaporation in high vacuum of ~10^−6^ mbar. Macroscopic top platinum electrode was also deposited by electron beam using GIS (Gas Injection System) attachment in FIB (Focused Ion beam) system. The voltage and current parameters for electron beam deposition was 10 kV and 1.7 nA, respectively. For the characterization of Cu+ immigration under the electric field, bulk CIPS (~15 μm thickness) sandwiched between two gold electrodes was prepared and the cross-sectional areas are exposed for the SEM-EDS measurement immediately after removing the bias.

### Scanning probe microscopy measurement

Vector PFM and c-AFM measurements were performed using a commercial atomic force microscope (Asylum Research MFP-3D) with Pt/Ir-coated Si cantilever tips with a radius of ~25 nm and spring constants of ~0.5–9.5 N/m. In vector PFM mode, ferroelectric domains were imaged by the tip driven with an ac voltage (*V*_ac_ = 0.5–1 V). The PFM was acquired at the drive frequency of ≈350 kHz in Vector PFM mode. In c-AFM measurement, single point current–voltage (*I*–*V*) measurements were performed with ORCA module with compliance current of 20 nA. The voltage was applied to the conducting Au bottom electrode, which was continuously swept to simultaneously read the current. The precise tip force was calculated by multiplying the spring constant, the inverse optical lever sensitivity (InvOLS), and setpoint. All these *I*–*V* measurements were performed at ambient temperature.

### Simulation and calculation

#### *I*–*V* simulation

The electrical measurement setup was described by a circuit model for the AFM tip/CIPS flake/Au substrate. The tip-flake and flake-substrate interfaces were represented as Schottky barriers connected in a back-to-back geometry. A resistance, R, connected in series between the two Schottky barriers accounts for bulk resistance of the CIPS flake. The modified thermionic emission theory is described by^34^:

1 $${{I}}\left(V\right)={I}_{0}{{\exp }}^{\left(-\beta V/{k}_{B}T\right)}\left({{\exp }}^{\left({qV}/{k}_{B}T\right)}-1\right)$$

2 $${I}_{0}={A}_{c}{A}^{* }{T}^{2}{{\exp }}^{\left(-q{\varphi }_{b}/{k}_{B}T\right)}$$

where *I*_0_ is the saturation current of the junction, *q* is the electron charge, *V* is the applied voltage, *k*_B_ is the Boltzmann constant, *T* is the temperature, *A*_c_ is the Schottky barrier contact area, A^*^ is the effective Richardson constant, and *φ*_b_ is the initial energy of the Schottky barrier, *β* factor is introduced to mimic the influence of mobile Cu^+^ ion on the profiles of *I*–*V* curves and mainly describes the degree of Cu^+^ ions migration in different voltage direction.

#### Strain and strain gradient calculation

The strain and strain gradient distributions in CIPS flakes were obtained from theoretical simulations with a tip-force model under a tip force of 4.2 μN^31^. The Pt/Ir-coated silicon tip was considered as the rigid sphere, the Young’s modulus and Poisson’s ratio of CIPS are 25 GPa and −0.06, respectively^23,35^. The piezoelectric and flexoelectric fields were obtained by using the linear piezoelectric equation and the flexoelectric constitutive equation, respectively. The surface potentials induced by piezoelectric and flexoelectric effects were obtained by integrating the piezoelectric field and flexoelectric field. The details of calculations can be found in supplementary materials.

#### Density functional theory calculation

In this work, the effect of vertical strain on bandgap of CIPS was investigated using the Vienna Ab initio Simulation Package (VASP)^36,37^ based on the density functional theory (DFT). The electron-ion interactions were described by Projector-augmented-wave (PAW) potentials^38^, while Perdew-Burke-Ernzerhof functional of generalized gradient approximation^39^ was chosen to deal with the exchange-correlation functional. The cut-off energy of 450 eV was for the plane-wave basis and the Monkhorst-Pack mesh^40^ with k-spacing of 0.2 Å^−1^ was set for k-point sampling. And the energy and force convergence criterions were 10^−6^ eV and 10^−3^ eV/Å^−1^, respectively. In order to take into account the interlayer van der Waals interaction in bulk CIPS, DFT-D2 method^41^ was adopted.

## Supplementary information

Supplementary Information

## Data Availability

The data that support the findings of this study are available from the corresponding author on request.
