# Supplementary material for: Manipulation of current rectification in van der Waals ferroionic CuInP2S6
Source: Nat Commun. 2022 Jan 31;13:574. doi: 10.1038/s41467-022-28235-6 (PMC8803863; doi:10.1038/s41467-022-28235-6)
Supplement: Supplementary file 1 — Supplementary Information [file 41467_2022_28235_MOESM1_ESM.pdf]

Supplementary Materials for

Manipulation of Current Rectification in Van der Waals ferroionic $CuInP_2S_6$

Xingan Jiang[1], Xueyun Wang[1*], Xiaolei Wang[2*], Xiangping Zhang[1], Ruirui Niu[3], Jianming Deng[1], Sheng Xu[4], Yingzhuo Lun[1], Yanyu Liu[1,5], Tianlong Xia[4], Jianming Lu[3], and Jiawang Hong[1*]

[1]School of Aerospace Engineering, Beijing Institute of Technology, Beijing, 100081, China

[2]College of Physics and Optoelectronics, Faculty of Science, Beijing University of Technology, Beijing 100124, China

[3]State Key Laboratory for Mesoscopic Physics and Frontiers Science Center for Nano-optoelectronics, School of Physics, Peking University, Beijing 100871, China

[4]Department of Physics and Beijing Key Laboratory of Opto-electronic Functional Materials & Micro-nano Devices, Renmin University of China, Beijing 100871, China

[5]College of Physics and Materials Science, Tianjin Normal University, Tianjin 300387, PR China

*Corresponding Authors Emails: Xueyun Wang (xueyun@bit.edu.cn), Xiaolei Wang(xiaoleiwang@bjut.edu.cn), Jiawang Hong (hongjw@bit.edu.cn)

Supplementary Figures:

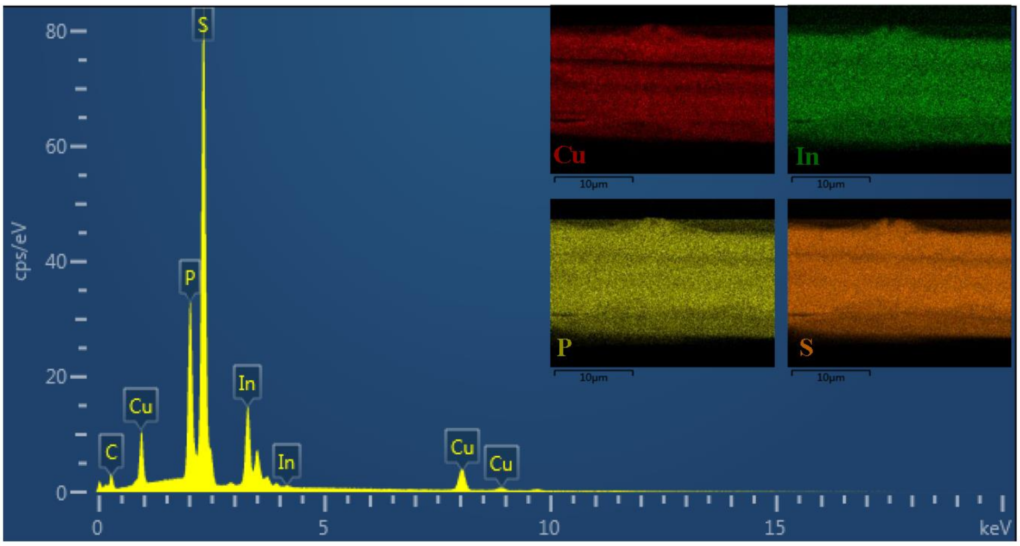

Supplementary Fig. 1. EDS mapping of cross-sectional CIPS single crystal. Cu, In, P and S elements are uniformly distributed in the bulk CIPS.

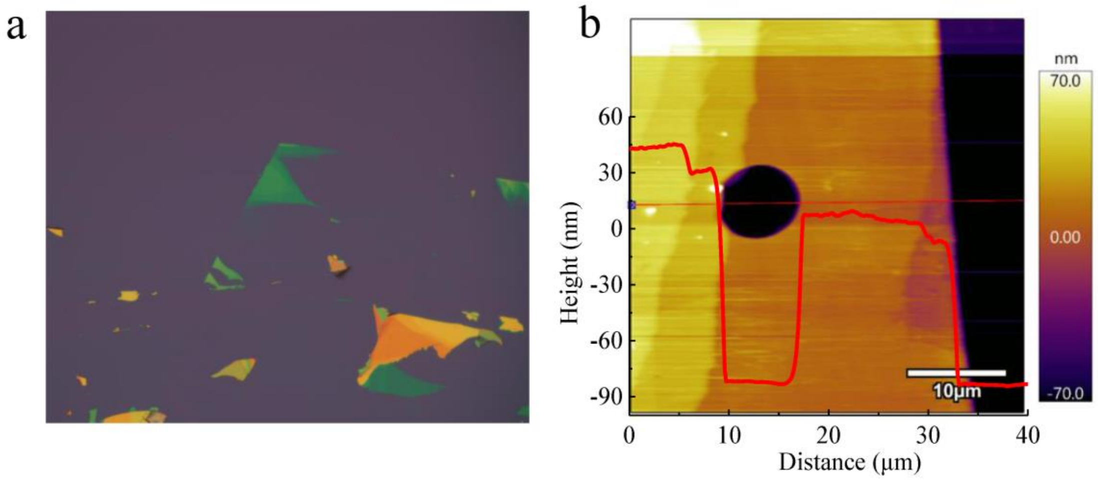

Supplementary Fig. 2. The exfoliated CIPS flakes by the observation of optical microscopy and AFM characterization. a Optical microscopy of CIPS flakes on Au conducting substrate; **b** The topography of CIPS flakes measured by atomic force microscopy in tapping mode. The thickness of CIPS flake can be precisely obtained by measuring the height difference in the line profile between CIPS flakes and the substrate. The thickness of CIPS flakes ranges from 80 to 120 nm.

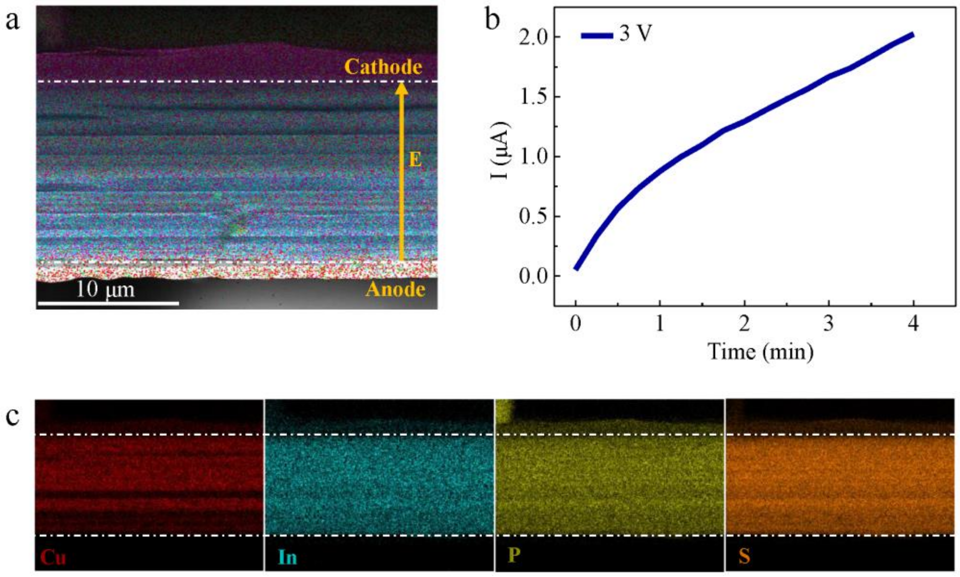

Supplementary Fig. 3. The electric field driven Cu^+ ionic migration. a The cross-sectional view of bulk CIPS (~15 μm) sandwiched between two gold electrodes for the EDS measurement; **b** The time dependent current at an external bias voltage 3V, the higher current with the longer time is indicative of time-dependent Cu^+ migration; **c** The EDS is measured immediately after removing the bias. The dotted line represents the interface between material and electrode. It is clear that In, P and S elements remain evenly distributed, but the Cu^+ ions show clear migration towards cathode side and thus gives unevenly distribution in the bulk, leading to Cu element abundance near cathode and Cu deficiency near anode.

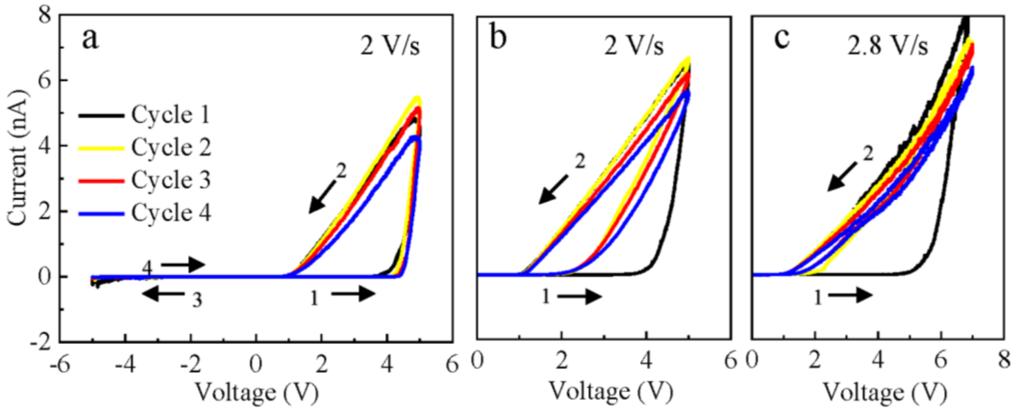

Supplementary Fig. 4. I-V curves for four consecutive cycles with different voltage sweeping. **a**, 0 V→+5 V→-5 V→0 V; **b**, 0 V→+5 V→0 V; c, 0 V→+7 V→0 V. One full cycle was completed in 10 second, where the sweeping speed is 2 V/s for **a, b** and 2.8 V/s for **c**.

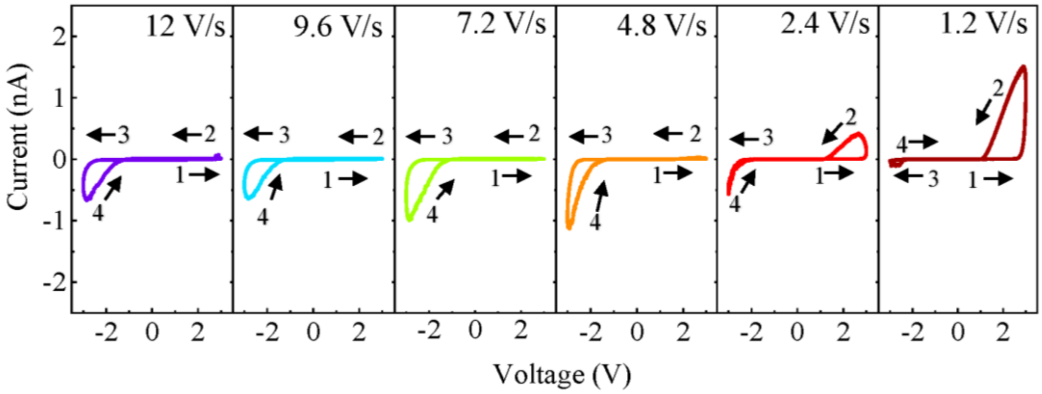

Supplementary Fig. 5. A series of full cycle of I-V curves consecutively measured under a voltage sweeping of 0 V→+3 V→-3 V→0 V with a gradually slower sweeping speed ranging from 12 V/s to 1.2 V/s. Note that the number 1-4 denotes the sweeping sequence in full cycle.

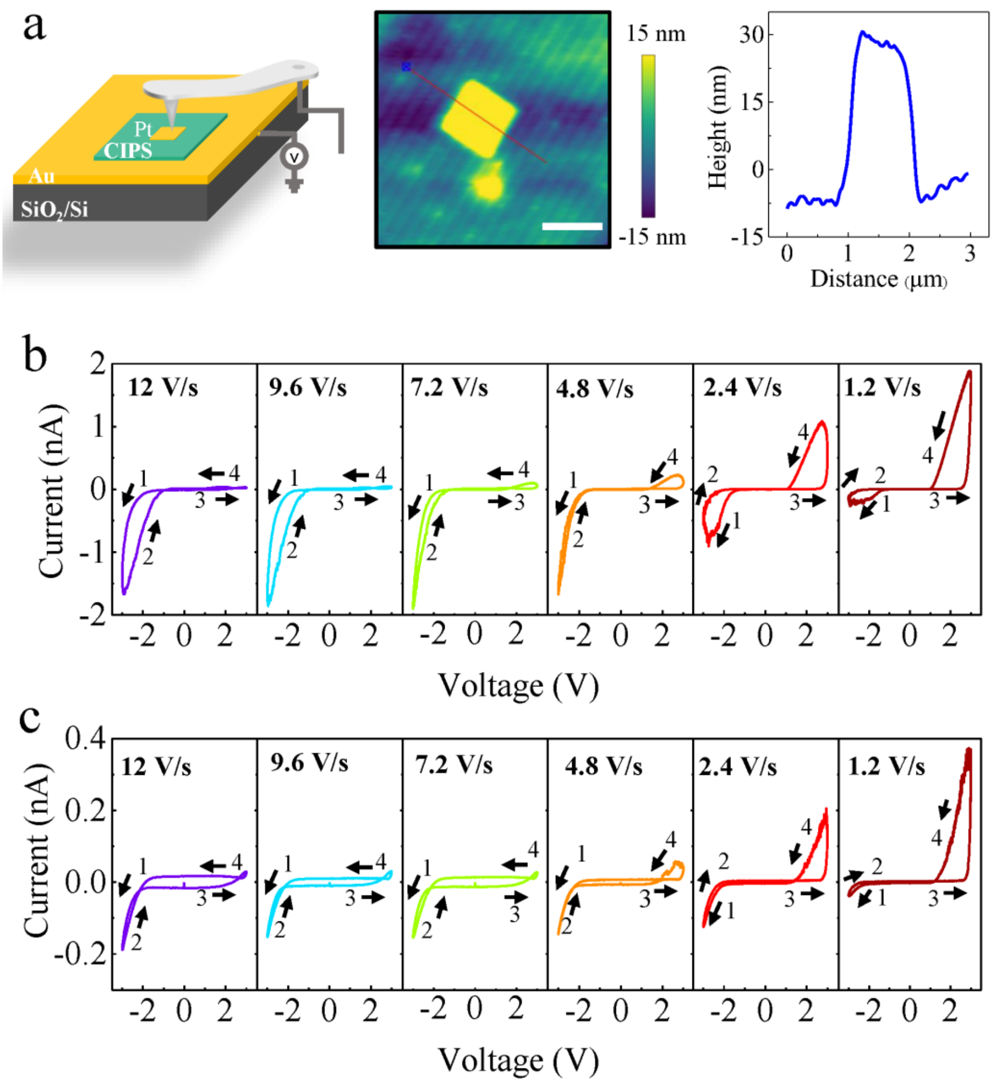

Supplementary Fig. 6. The electrical measurement using different contact electrode. a The schematic electrical measurement setup, the topography of CIPS flake with top Pt electrode and the height line profile across the Pt electrode; The scale bar is 1 μm. **b** The obtained I-V curves by using AFM tip directly in contact with the surface of CIPS flake; **c** The obtained I-V curves by using the top Pt electrode. I-V curves are consecutively measured at a sweeping speed ranging from 12 V/s to 1.2 V/s. The numbers 1-4 denote the sweeping sequence.

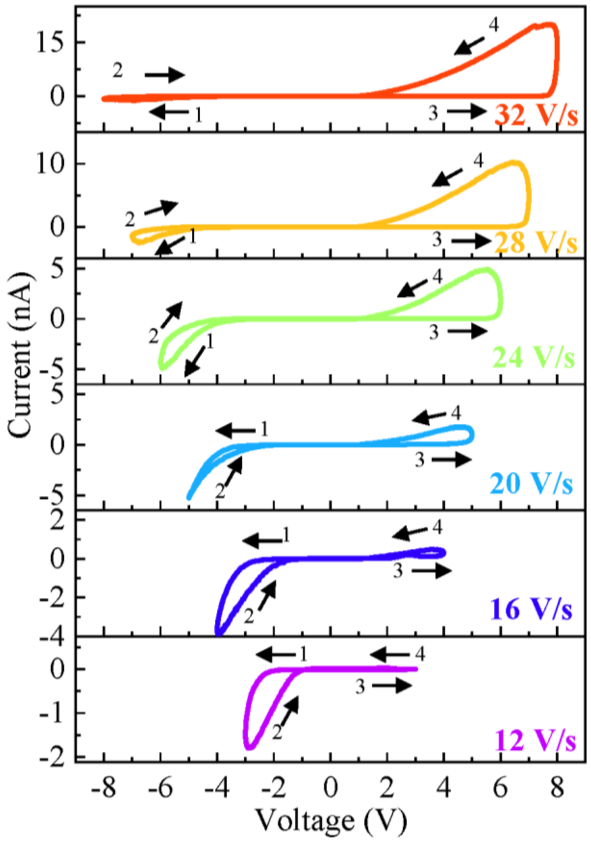

Supplementary Fig. 7. The voltage dependent current-voltage (I-V) full-cycles measured consecutively with a voltage sweeping cycle (0 V→-V_{max}→+V_{max}→0 V). Note that the number 1-4 denotes the sweeping sequence. The value of V_{max} ranges from 3 V to 8 V and the interval is 1 V. One full cycle was completed in 1 second, where the sweeping speed is 12, 16, 20, 24, 28, 32 V/s.

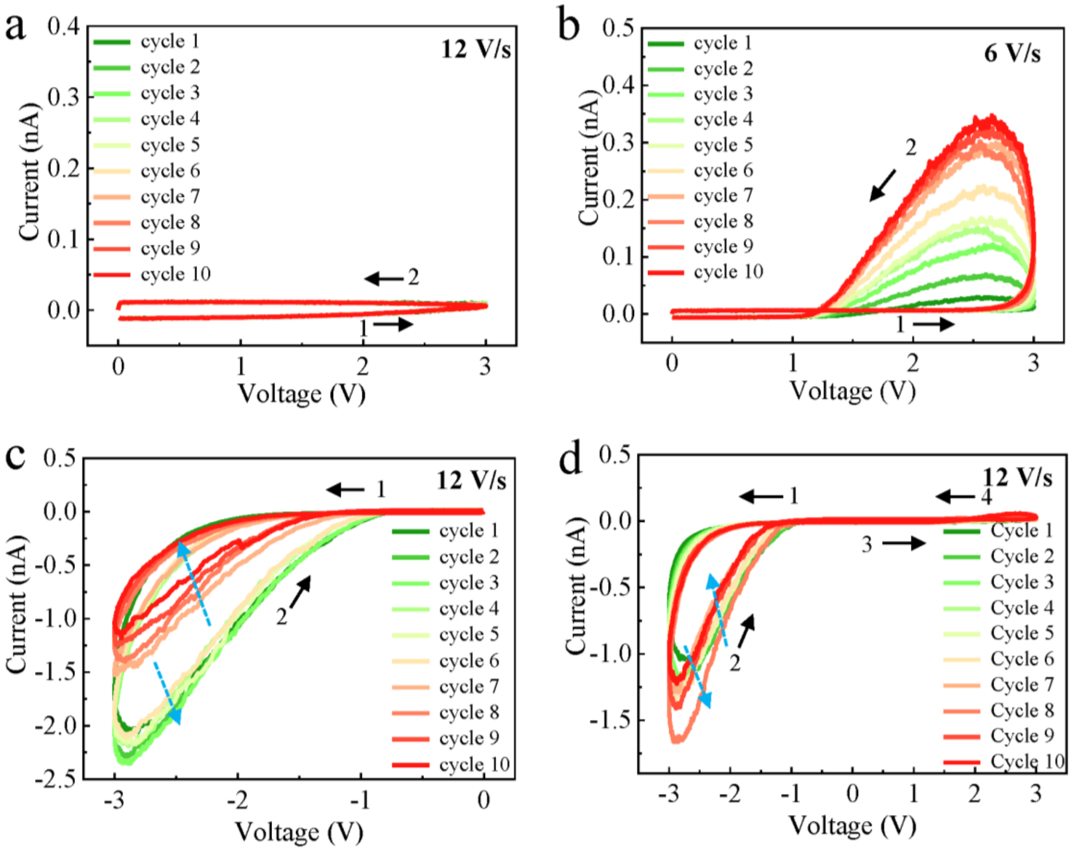

Supplementary Fig. 8. I-V curves consecutively measured with a positive/negative voltage sweeping. a, **b** The semi-cycle of I-V curves consecutively measured with a positive voltage sweeping of 0 V→+3 V→0 V with 12 V/s and 6 V/s sweeping speed, respectively; **c** The semi-cycle of I-V curves consecutively measured with a negative voltage sweeping of 0 V→-3 V→0 V at a 12 V/s sweeping speed; **d** The full-cycle of I-V curves consecutively measured with a voltage sweeping of 0 V→-3 V→ +3 V→0 V at a 12 V/s sweeping speed. The number 1-4 denotes the sweeping sequence.

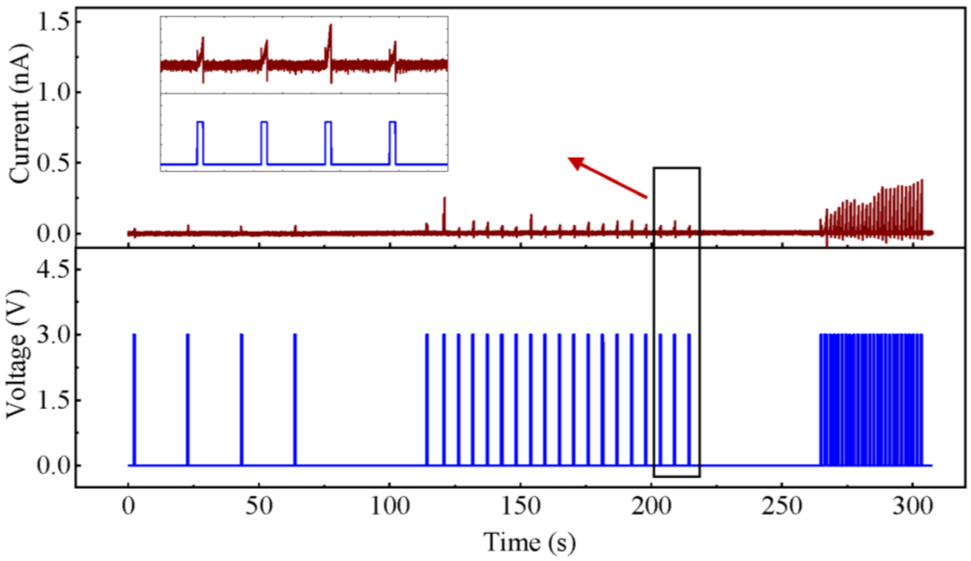

Supplementary Fig. 9. The electrical results of CIPS flake under three sets of 3 V pulse voltage with different pulse intervals. The pulse interval are 20 s, 5 s, and 1 s, respectively. The pulse voltage width is 0.5 s. The inset shows the enlarged view of the pulse signal and detected current in the black box region.

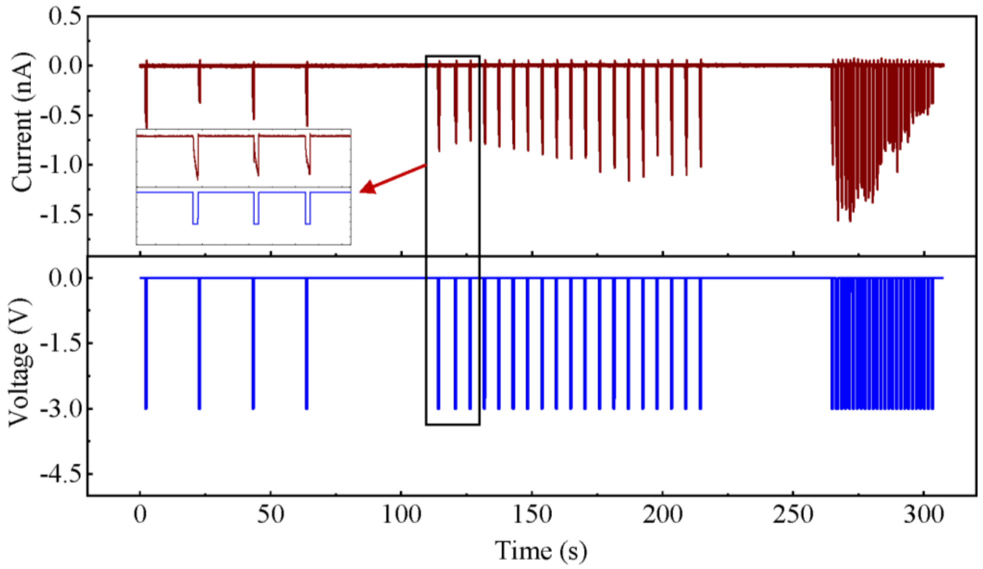

Supplementary Fig. 10. The electrical results of CIPS flake under three sets of -3 V pulse voltage with different pulse intervals. The pulse interval are 20 s, 5 s, and 1 s, respectively. The pulse voltage width is 0.5 s. The inset shows the enlarged view of the pulse signal and detected current in the black box.

Supplementary Note 1. The schematic circuit model and the I-V curve simulation

Our experimental setup can be described by a circuit model for the AFM tip/CIPS flake /Au substrate, as shown in **Supplementary Fig. 11**. The ideal rectifier characteristic of Schottky barriers can be described according to the ideal thermionic emission theory, the current I through a ideal Schottky barrier is given by[1]:

$$I(V) = I_0 \left(exp^{(qV/k_BT)} - 1 \right) \quad (1)$$

$$I_0 = A_c A^* T^2 exp^{(-q\varphi_b/k_BT)} \quad (2)$$

where I_0 is the saturation current of the junction, q is the electron charge, V is the applied voltage, k_B is the Boltzmann constant, T is the temperature, A_c is the Schottky barrier contact area, A^* is the effective Richardson constant and φ_b is the initial energy of the Schottky barrier. However, this form does not apply in the case of Cu^+ ions migration and redistribution. The Cu^+ ions redistribution significantly modifies the Schottky barrier, which affects the current rectification. Herein, we further use the modified thermionic emission theory to describe the electron transfer at the terminal interface, and use β factor to fully consider the influence of Cu ion redistribution on the two terminal Schottky barrier and modify the I-V rectification due to Cu ions migration. The modified thermionic emission theory is described by:

$$I(V) = I_0\, exp^{(-\beta qV/k_BT)} \left[exp^{(qV/k_BT)} - 1 \right] \quad (3)$$

Therefore, β factor considers the influence of Cu ion redistribution on the Schottky barrier and modify the I-V rectification. We extracted the I-V experimental data in high conducting state in **Fig. 4b** and use the MATLAB to solve the circuit and simulate the I-V curves. The interface effect in this system is dominated by the AFM tip/CIPS contact. As expected in **Supplementary Fig. 12**, we found that the rectifying behavior of the I-V curves is dominated by the tip/CIPS contact because the profile and rectification of the I-V curves show negligible changes as we treat CIPS/Au substrate as the ohmic contact.

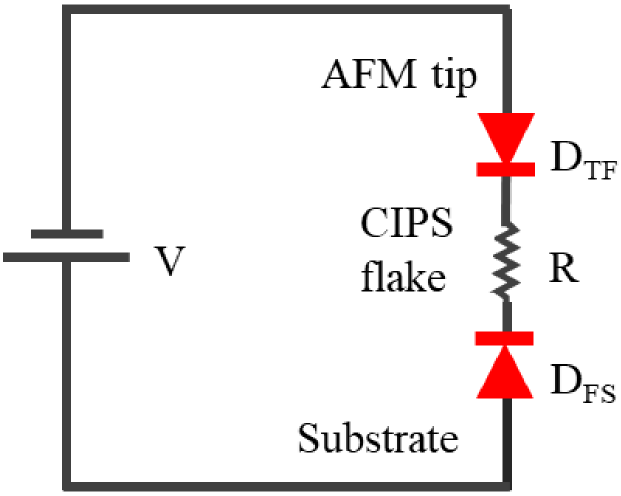

Supplementary Fig. 11. The schematic circuit model for the AFM Tip/CIPS flake /Au substrate in our experimental setup. The tip-flake and flake-substrate interfaces are represented as Schottky barriers connected in a back-to-back geometry. A resistance, R, connected in series between the two Schottky barrier accounts for the bulk resistance of the CIPS flake.

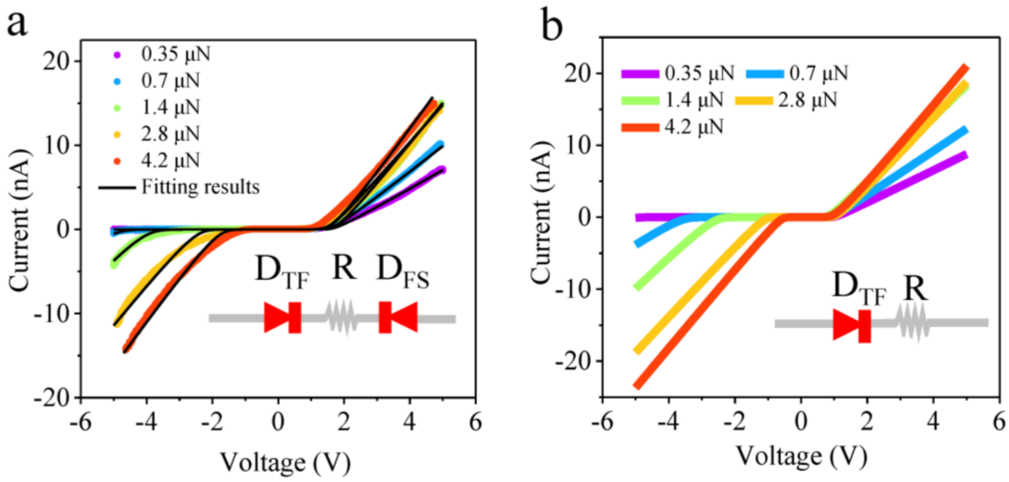

Supplementary Fig. 12. The rectifying behavior of the I-V curves dominated by the tip/CIPS contact. a The experimental and simulated I-V curves under different tip force using D_{TF}/R/D_{FS} circuit model; **b** The simulated I-V curves under different tip force without considering the Au/Substrate Schottky barrier (treating CIPS/Au contact as the ohmic contact).

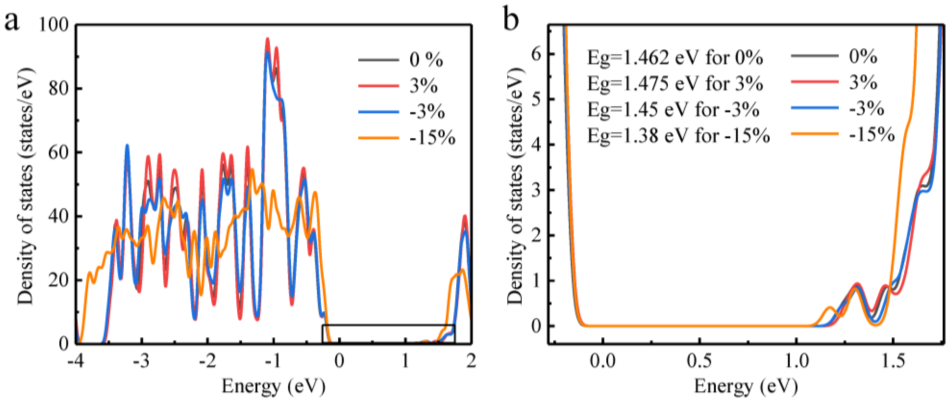

Supplementary Fig. 13. Strain induced bandgap change for CIPS by DFT calculation. a DFT calculation results of density of states under different strain for CIPS; **b** the magnification corresponding to the black rectangular box in **a**. Under a tip force of 4.2 μN, the compressive strain in the local area can reach up to 5-15%**.** The band gap only slightly decreases by ~0.1 eV under 15% compressive strain, suggesting the band gap change is not the dominating factor of Schottky barrier change under the tip force.

Supplementary Note 2. The estimated contact area using Hertzian contact model.

The contact area varies with the tip force using the Hertz contact model[3], as shown in **Supplementary Fig. 14**. The contact area increases by a factor of ~7, from 150 nm^2 to 1008 nm^2 as the force increases from 0.35 μN to 4.2 μN, which cannot explain the enhanced current of several orders of magnitude in negative voltage direction.

AFM tip is modeled as a sphere of radius R and the flake as a half space. The load force F and the indentation depth d are related by[2] :

$$F = \frac{4}{3} E^* R^{\frac{1}{2}} d^{\frac{3}{2}} \tag{4}$$

where R is the curvature radius of the AFM tip (~25 nm) and E^* is a function of the Young moduli (E_T and E_F), and the Poisson ratios (v_T and v_F) of the silicon tip and the CIPS flake, respectively:

$$\frac{1}{E} = \frac{1-{v_T}^2}{E_T} + \frac{1-v_F^2}{E_F} \tag{5}$$

The radius of the contact area is related with the applied force by:

$$a = \left(\frac{3FR}{4E^*}\right)^{\frac{1}{3}} \tag{6}$$

and the tip-flake contact area A_C is

$$A_C = \pi\left(a^2 + d^2\right) = \pi\left\{\left(\frac{3FR}{4E^*}\right)^{2/3} + \frac{1}{R^2}\left(\frac{3FR}{4E^*}\right)^{4/3}\right\} \tag{7}$$

Young moduli (E_T and E_F) , and the Poisson ratios (v_T and v_F) of the silicon tip and the CIPS flake is E_T = 180 GPa, and E_F = 25 GPa, v_T = 0.2, and v_F = -0.06, respectively[2-4].

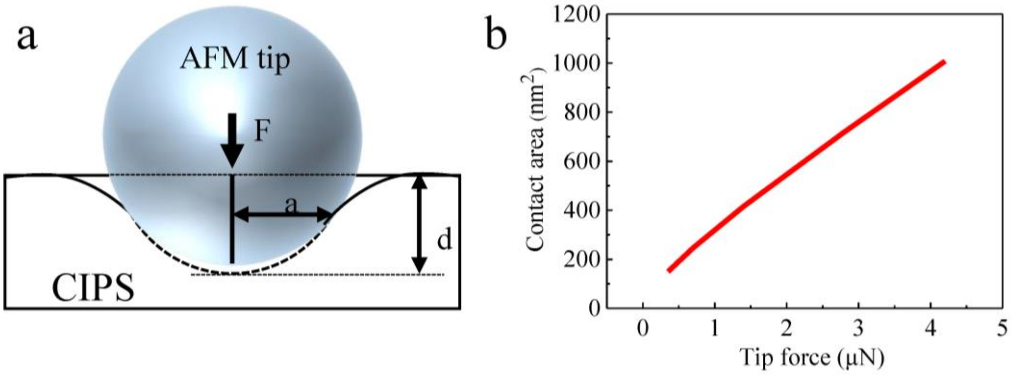

Supplementary Fig. 14. The estimated contact area using Hertzian contact model. **a** Hertzian contact model. The AFM tip is modeled as a sphere and the CIPS flake as a semi-space. F is the applied load force, a is the radius of the contact area and d is the indentation depth; **b** Tip-flake contact area change as a function of the tip force.

Supplementary Note 3. The quantitatively analysis of the barrier height modulation due to strain or strain gradient.

Based on the Hertz contact theory model, the tip is treated approximately as a hemispherical rigid body, and the indentation deformation is regarded as a pure elastic process under the control of classical continuum mechanics. The analytical strain field under spherical indenter in Hertz contact theory has been widely developed[5,6]. The strain gradient field distribution can be obtained by taking the spatial derivative of the analytical solution. In cylindrical coordinates, the elastic stress field in a semi-infinite block is as follows[7]:

$$\frac{\sigma_{rr}}{p_m}=\frac{3}{2}\left\{\frac{1-2v}{3}\frac{a^2}{r^2}\left[1-\left(\frac{z}{u^{1/2}}\right)^3\right]+\left(\frac{z}{u^{1/2}}\right)^3\frac{a^2u}{u^2+a^2z^2}\right.$$
$$\left.+\frac{z}{u^{1/2}}\left[u\frac{1-v}{a^2+u}+(1+v)\frac{u^{1/2}}{a}\tan^{-1}\left(\frac{a}{u^{1/2}}\right)-2\right]\right\} \tag{8}$$

$$\frac{\sigma_{\theta\theta}}{p_m}=-\frac{3}{2}\left\{\frac{1-2v}{3}\frac{a^2}{r^2}\left[1-\left(\frac{z}{u^{1/2}}\right)^3\right]\right.$$
$$\left.+\frac{z}{u^{1/2}}\left[2v+u\frac{1-v}{a^2+u}-(1+v)\frac{u^{1/2}}{a}\tan^{-1}\left(\frac{a}{u^{1/2}}\right)\right]\right\} \tag{9}$$

$$\frac{\sigma_{zz}}{p_m}=-\frac{3}{2}\left(\frac{z}{u^{1/2}}\right)^3\left(\frac{a^2u}{u^2+a^2z^2}\right) \tag{10}$$

$$\frac{\tau_{rz}}{p_m}=-\frac{3}{2}\left(\frac{rz^2}{u^2+a^2z^2}\right)\left(\frac{a^2u^{1/2}}{a^2+u}\right) \tag{11}$$

where

$$u=\frac{1}{2}\left[\left(r^2+z^2-a^2\right)+\left[\left(r^2+z^2-a^2\right)^2+4a^2z^2\right]^{1/2}\right] \tag{12}$$

$$p_m=\frac{F}{\pi a^2} \tag{13}$$

$$a=\left(\frac{3}{4}\frac{FR}{E}\left(1-v^2\right)\right)^{\frac{1}{3}} \tag{14}$$

$$\frac{1}{E}=\frac{1-v^2}{E_S}+\frac{1-v_{tip}^2}{E_{tip}} \tag{15}$$

In the above expression, F represents the force applied to the probe (4.2 μN), R is the tip radius (25 nm), A is the contact radius, E is the effective Young's modulus, V is Poisson's ratio, and both r and z are cylindrical coordinates. The above expression does not depend on θ because of radial symmetry.

The flexoelectric effect-induced electric field is linearly proportional to the flexoelectric coupling coefficient. However, there is no reported value of flexoelectric coefficient of CIPS so far. Here, the flexoelectric coupling coefficients in the longitudinal, transverse and shear direction are considered to be -6V, -3V and -2V in the following calculation, respectively, which is in the range of the flexoelectric coupling coefficient of ±1-10 V[5,8]. The piezoelectric properties of CIPS have been studied extensively, so we refer to the piezoelectric coefficients reported in the recent works[3, 9]. The strain and strain gradient distribution under a tip force of 4.2 μN are shown in **Supplementary Fig. 15a and b**, respectively. The calculated surface potentials induced by the piezoelectric/flexoelectric effect are shown in **Supplementary Fig. 16**.

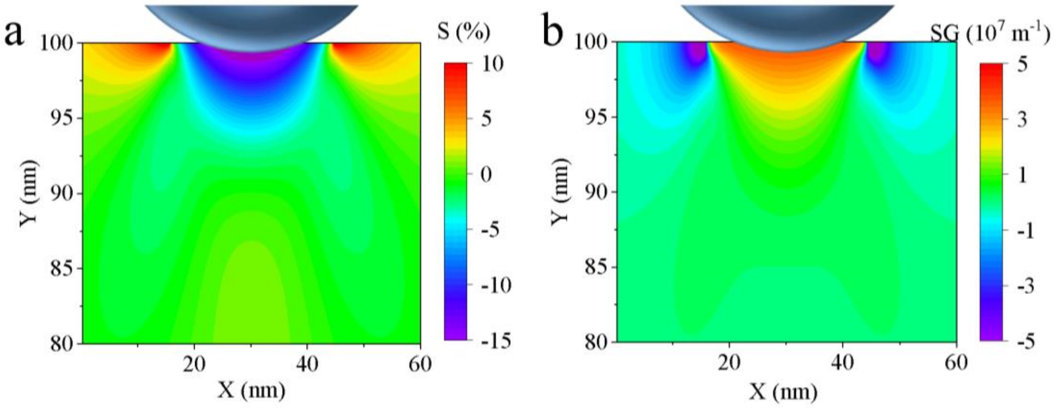

Supplementary Fig. 15. The strain (a) and strain gradient (b) distribution in CIPS flakes obtained from theoretical calculations with a tip-force model under a tip force of 4.2 μN. The tip is treated approximately as a hemispherical rigid body with a radius of 25 nm, and the indentation deformation is regarded as a pure elastic process under the control of classical continuum mechanics.

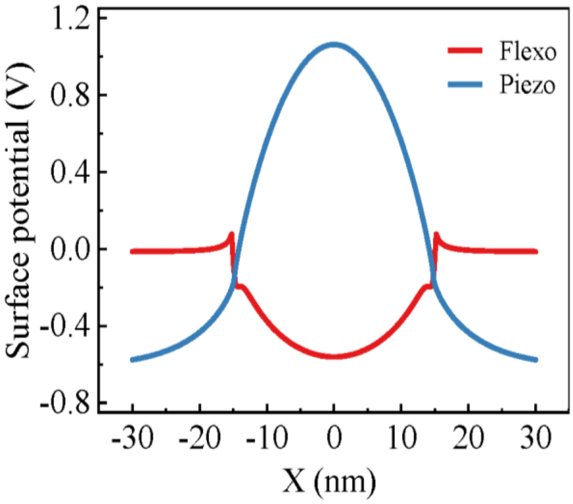

Supplementary Fig. 16. The calculated surface potential induced by piezoelectric/flexoelectric effect under the tip force of 4.2 µN.

References

1. Rhoderick, E. H. & Rothwarf, A. Metal-semiconductor contacts. *Phys. Today*. **32**, 66-71 (1979).
2. Yang, M. M., Kim, D. J. & Alexe, M. Flexo-photovoltaic effect. *Science*. **360**, n3256 (2018).
3. You, L. et al. Origin of giant negative piezoelectricity in a layered van der Waals ferroelectric. *Sci. Adv*. **5**, eaav3780 (2019).
4. Zhang, C., Nie, Y., Aijun, D. U. & Chemistry, S. O. Intrinsic Ultrahigh Negative Poisson's Ratio in Two-Dimensional Ferroelectric ABP_2X_6 Materials. *Acta. Phys. Chim. Sin.* **32**, 1128-1133 (2019).
5. Mizzi, C. A., Lin, A. and Marks, L. D, Does Flexoelectricity Drive Triboelectricity?. *Phys. Rev. Lett*. **123**, 116103 (2019).
6. Wang, L. et al. Flexoelectronics of centrosymmetric semiconductors. *Nat. Nanotechnol.* **15**, 661-667 (2020).

7. Wriggers, P. Introduction to Contact Mechanics. Springer US, 2007.

8. Shi, W., Y Guo., Zhang, Z, et al. Flexoelectricity in Monolayer Transition Metal Dichalcogenides. *J. Phys. Chem. Lett*. **9**, 6841–6846 (2018).

9. Neumayer, S. M., Eliseev, E, A., Susner, M. A, et al. Giant negative electrostriction and dielectric tunability in a van der Waals layered ferroelectric. *Phys. Rev. Mater.* **3**, 024401 (2019).
